# Partially Treated Acute Suppurative Thyroiditis in a Healthy Child Caused by Streptococcus pyogenes

**DOI:** 10.7759/cureus.39227

**Published:** 2023-05-19

**Authors:** Haitham Almoffarreh, Nouf Alkaabi, Abdullatif Alkhurayji

**Affiliations:** 1 Pediatric Emergency Medicine, King Abdullah Specialist Children’s Hospital, King Abdulaziz Medical City, Riyadh, SAU

**Keywords:** infection, streptococcus pyogenes, abscess, thyroid gland, parapharyngeal

## Abstract

Thyroid abscess is a rare encounter due to its unique histological and structural features. It is usually associated with some form of congenital anomalies in pediatrics especially if recurred. Early recognition and treatment are of paramount importance in preventing complications. Atypical presentation can occur if the patient is improperly treated prior to presentation. Conservative management is becoming the mainstay of treatment unless there is a risk of airway compromise or extension.

We report the case of a 15-month-old female who presented with anterior neck swelling. She received oral antibiotics prior to her visit and did not exhibit severe systemic illness despite her disease extension. She was found to have a thyroid abscess originating from the left thyroid lobe extending to the mediastinum. No congenital anomalies were found. She was managed with open drainage and her cultures grew Streptococcus pyogenes.

## Introduction

A thyroid abscess is a rare condition and uncommon complication of neck infection in the pediatric age group [1,2] due to the anatomical and physiological characteristics of the thyroid gland in resistance to infection [3]. Infection can occur as a result of direct extension in the neck region or from inoculation due to direct trauma. However, the most likely source is via hematogenous spread from a remote infection. The most common organisms involved in thyroid abscesses are *Staphylococcus* and *Streptococcus* species, yet most infections are polymicrobial, and the flora varies widely depending on the patient’s immune status [4].

## Case presentation

A 15-month-old female, medically and surgically free, vaccinated up to nine months of age, presented to the emergency department complaining of neck swelling noticed by her parents for the past three days. The swelling was increasing in size and was associated with decreased feeding, voice hoarseness, and a weak cry. These symptoms were preceded by a history of pharyngitis, fever, and cough for which she received a five-day course of oral antibiotics, namely, amoxicillin/clavulanic acid for one week prior to presentation. After one day of discontinuing the antibiotics, the patient developed a fever again with a recurrence of her symptoms.

Physical examination revealed an awake child with obvious midline neck swelling, she was not in pain, with no trismus nor drooling. There was no audible stridor, normal swallowing, and no signs of any respiratory distress. A local neck exam showed a 3 x 4 cm firm anterior midline swelling with minimal fluctuation and extending to the left para-tracheal side. The overlying skin was erythematous and non-tender with no discharge. There were no palpable lymph nodes, and the rest of her physical exam was unremarkable.

In the emergency department, the patient was kept nil per oral and started on intravenous dextrose 5% normal saline with an empirical dose of intravenous clindamycin. Her blood work showed normal thyroid function, leukocytosis with a white blood cell count of 16.30 x 10^9/L, a high neutrophil count of 11.00 x 10^9/L, and a high platelet count of 1179 x 10^9/L. She also had high inflammatory markers including a c-reactive protein of 44 mg/dl and an erythrocyte sedimentation rate of 117 mm/hr.

**Figure 1:**
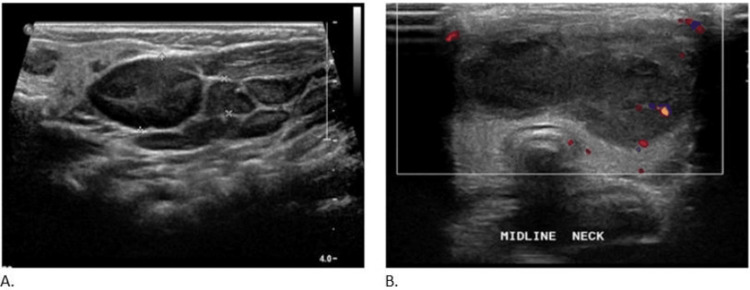
(A) Left lateral ultrasound and (B) midline neck ultrasound The images show an anterior midline neck collection which is multi-septated with thick walls and internal debris. It measures 5.5 x 1.5 x 4 cm with a total volume of 17.5 ml.

A neck ultrasound was showing an anterior midline neck collection which is multi-septated with thick walls and internal debris. It measures 5.5 x 1.5 x 4 cm with a total volume of 17.5 ml (Figures 1A-1B).

**Figure 2:**
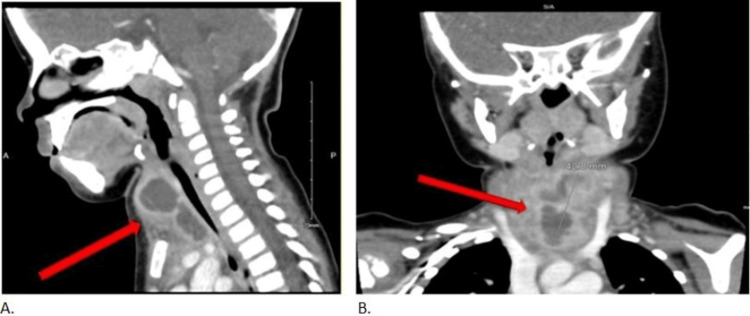
(A) Sagittal view and (B) coronal view CT of the neck The images show an anterior midline neck collection that is multi-septated with thick enhancing irregular walls extending to the superior mediastinum, with no evidence of significant airway narrowing.

A neck CT was showing an anterior midline neck collection that is multi-septated with thick enhancing irregular walls extending to the superior mediastinum. It is inseparable from the thyroid gland and likely originating from the left thyroid lobe and measures 3.4 x 2.8 x 4.3 cm in the transverse, anterior-posterior, and craniocaudal dimensions, respectively, with no evidence of significant airway narrowing (Figure 2).

The patient was admitted to the hospital for open incision and drainage using Penrose with the ENT team under general anesthesia. Fluid culture from the drained abscess came positive for *Streptococcus pyogenes* (Group A). It was also sent for fungal and acid-fast bacilli cultures which came back to be negative and negative tuberculosis polymerase chain reaction. The patient improved, her symptoms were relieved, and the swelling subsided. She was discharged home with oral amoxicillin/clavulanic acid for three weeks and a follow-up in the clinic.

**Figure 3:**
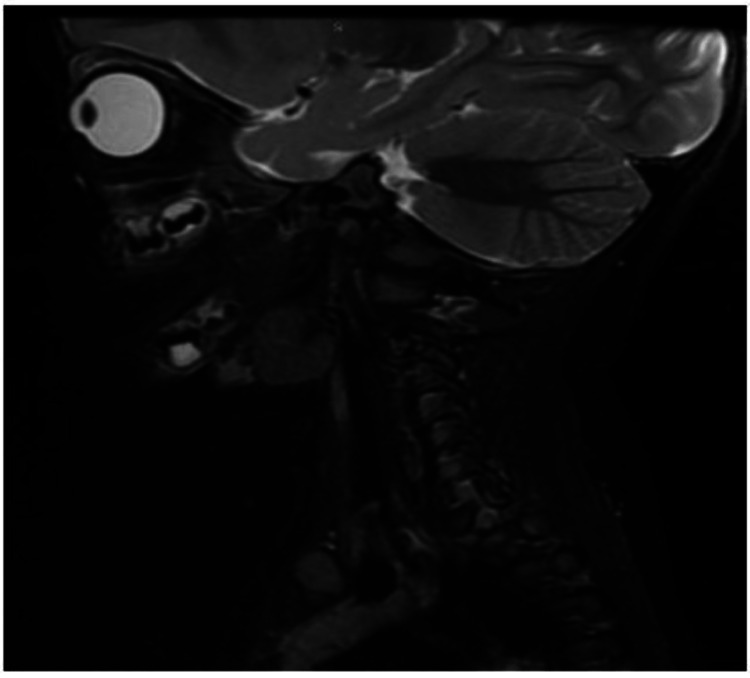
Sagittal view MRI of the neck The image shows complete resolution of the abscess with no evidence of collections or pathological lymph nodes and no anatomical defects.

The patient was followed up in outpatient settings by ENT and infectious diseases teams. Her condition significantly improved, and her inflammatory markers normalized. A neck MRI was done as an outpatient and reported no evidence of collections, no pathological lymph nodes, and no anatomical defects (Figure 3).

## Discussion

The thyroid gland has unique structural and histological features that render it resistant to infections. It has an encapsulation, rich lymphatic drainage, dual blood supply, and no communication to the surrounding structures, which makes the extension of infection unlikely [5].

Due to that, acute suppurative thyroiditis is considered rare and accounts for about 0.1%-0.7% [6]. The common background is a patient with immunosuppression and a patient with a pyriform sinus fistula. In the pediatric age group, a preceding upper respiratory tract infection or otitis media is common [7,8].

Most isolated organisms in pediatrics with acute suppurative thyroiditis were gram-positive aerobes followed by gram-negative aerobes. Fungi, anaerobes, and mycobacteria were less common. Of the gram-positive aerobes, *Staphylococcus aureus*, *Staphylococcus epidermidis*, and *Streptococcus pneumoniae* were dominant [9]. Anatomically, the left lobe of the thyroid is most commonly affected in pediatric patients, and it has been hypothesized that it may reflect an anatomical abnormality such as a tract extending from the left pyriform fossa [10].

Early recognition and management are important to prevent serious complications such as airway compromise, sepsis, or mediastinitis [11]. In our patient, the thyroid abscess was large, and it extended to the upper mediastinum, and if in case it ruptured, it may lead to mediastinitis which is one of the life-threatening complications.

The impact on thyroid function varies from euthyroid to hypothyroid and hyperthyroid and being euthyroid is the most reported. Being in euthyroid status was the most common in pediatrics following bacterial infections such as our patient [12].

When discussing the modality of diagnosis, there are two points to consider: first is to delineate the extent of the disease and its complications rapidly in the emergency department and second is to detect any underlying predisposing anomalies.

Ultrasound is considered the first-line imaging study to confirm the thyroid abscess due to the excellent visualization of the thyroid gland and the lack of ionizing radiation. It has been argued that CT scans and/or MRIs are better techniques for diagnosing thyroid abscesses as they provide more information regarding the extension and spread of the infection in addition to any predisposing anomalies such as pyriform sinus fistula [13,14].

Given the patient’s age, a CT scan and MRI might require sedation which will take time, and a CT scan will expose the patient to radiation. Ultrasound is a reasonable first-line imaging study in the emergency department as it can detect early signs before progression to abscess formation. Based on findings on ultrasound, a decision can be made to proceed to the next step in imaging such as a CT scan [5,15].

In our case, an ultrasound was done initially in the emergency department as the patient was stable. After the findings on ultrasound, we then proceeded to CT scan of the neck and upper chest to seek further information regarding the extension and origin of the abscess. As inflammation might obscure the findings of the pyriform fossa sinus, an MRI was done after drainage and resolution of the symptoms which demonstrated no anomalies [6,14].

Definitive diagnosis was reached using incision and drainage, and culture from aspirated fluid grew *Streptococcus pyogenes*, and no granulomas were found in the aspirate.

Acute uncomplicated suppurative thyroiditis can be managed conservatively with proper antibiotics or single fine needle aspiration and followed closely for any persistence or progression. Some cases were managed successfully with antibiotics alone but for a long duration of 55 days [16]. In case of complications or extension of the abscess as with our patient, open drainage is the way to go especially if there is a concern about airway compromise [6]. Needle aspiration with sonographic guidance has proved successful in a few reported cases, even following a single aspiration [17,18].

Our patient was treated with an inadequate dose and duration for respiratory tract infection, which led to partially treating the infection. This in turn affected her presentation to our care and masked the picture as she was non-toxic and not exhibiting much of symptoms, although her infection extended up to the mediastinum. This has been reported before by Wongphyat et al [15], but unlike our case, their patients later developed more signs.

## Conclusions

In conclusion, thyroid abscess is a rare encounter due to its unique histological and structural features. In pediatrics, it is usually associated with some form of a congenital anomaly such as pyriform fossa sinus especially if there is a recurrent infection. These anomalies might not be easy to diagnose during the acute phase. Early recognition and proper treatment are of paramount importance in preventing complications such as sepsis and airway compromise. Atypical presentation can happen or the patient will present with mild symptoms if improperly treated with antibiotics prior to presentation. Conservative management is becoming the mainstay of treatment unless there is a risk of airway compromise or extension.
